# Revealing the diversity of internal body temperature and panting response for feedlot cattle under environmental thermal stress

**DOI:** 10.1038/s41598-023-31801-7

**Published:** 2023-03-25

**Authors:** M. A. Islam, S. Lomax, A. K. Doughty, M. R. Islam, P. C. Thomson, C. E. F. Clark

**Affiliations:** 1 Livestock Production and Welfare Group, School of Life and Environmental Sciences, Sydney Institute of Agriculture, University of Sydney, Camden, New South Wales 2570 Australia; 2 Department of Dairy Science, Faculty of Animal Science and Veterinary Medicine, Patuakhali Science and Technology University, Dumki, Patuakhali 8602 Bangladesh; 3 Allflex Livestock Intelligence, Allflex Australia Pty Ltd., 33 Neumann Road, Capalaba, Queensland 4157 Australia; 4 Sydney School of Veterinary Science, University of Sydney, Camden, New South Wales 2570 Australia

**Keywords:** Biological techniques, Physiology, Environmental impact

## Abstract

Core body temperature (CBT) regulation is crucial for mammalian wellbeing and survival. Cattle pant to dissipate excess heat to regulate CBT when ambient conditions exceed thermoneutral zones. However, to date, neither the variability in cattle heat response, the lagged response of CBT to thermal indices, nor the diurnal patterns of thermal indices, CBT and panting have been reported in the literature. We decomposed thermal indices, CBT and panting time-series data for 99 feedlot heifers across three discrete heat events into diurnal, trend and residual components. Both raw and decomposed data were analysed to explore the lagged CBT and panting responses and the association between series. We show ambient thermal conditions impact CBT with a 1-h lag despite a lag of between 1.5 to 3 h from raw data. Average individual panting scores were used to identify heat-susceptible and heat-tolerant cattle. Heat-susceptible cattle showed greater CBT (*P* < 0.01) between 8:00 and 23:00 and greater panting duration (*P* < 0.05) between 10:00 and 18:00 than heat-tolerant cattle under the same thermal conditions and these variations followed a similar pattern despite differences in cattle breed. This new information enables targeted amelioration and selection of individuals against heat susceptibility.

## Introduction

Global atmospheric temperature is predicted to rise by more than 1.5 to 2 °C by the end of the twenty-first century, under all greenhouse gas (GHG) emission scenarios^1^. Alongside increased temperature, heatwave events will be more intense and longer^2,3^ with direct and indirect negative impacts on global livestock welfare, performance and productivity^4^. Cattle are more susceptible to heat stress than other livestock species due to their greater metabolic rate and reduced water retention capacity^5^, and are typically reared in extensive environments outdoors in most parts of the world. Cattle can regulate their core body temperature (CBT) in the range of 38 to 39 °C with a mean value of 38.6 ± 0.5 °C^6,7^; however, ambient thermal conditions beyond thermoneutral zones (5–25 °C)^8^ can impact cattle CBT regulation. If cattle fail to dissipate self-generated and absorbed heat energy into the surrounding atmosphere, CBT will rise, with the activation of evaporative heat dissipation mechanisms including increased sweating and panting^9,10^. Thus, elevated CBT is an effective indicator of heat stress^11–14^. However, CBT monitoring methods are invasive, and often are not suitable or practical for real-time and/or longer duration observations^15,16^. Further, time-series analyses considering auto-correlation and cross-correlation for continuously recorded CBT and temperature humidity index (THI)^17^ failed to reveal a causal relationship between absolute THI and the CBT of dairy cattle, although the relationship was significant at a THI threshold greater than 72 with a 2-h delayed response in CBT^18^. Therefore, heat stress monitoring and mitigation in cattle is based on visual assessment of animal response which consists of measuring breaths per minute (BPM)^19–21^ or panting score (PS). Panting score is evaluated on a scale of 0 to 4, where 0 indicates normal breathing and 4 indicates severe open-mouth breathing with tongue out^22–24^. Additionally, thermal indices are used to set thresholds for heat stress^23,25–28^. However, panting response to similar thermal conditions varies between time of day and individuals within a pen^29,30^. Cattle CBT also follows a circadian rhythm with a peak in the afternoon hours and a minimum in the morning^31^. Further work is required to determine the association between cattle panting severity and elevated CBT considering the common circadian rhythm of CBT, panting and thermal indices.

Routine visual monitoring and evaluation of individual farm animals to maintain animal wellbeing has reduced with the increased number of animals per operation^32–34^. Visual monitoring has limitations including, but not limited to, labour intensiveness, non-continuity of observation, bias, interference with the animal and subjectivity^35–37^. Most of the limitations of visual assessment have been omitted in thermal stress–strain indices like the THI^17^ or the more detailed heat load index (HLI)^23^ and the HLI is regularly used in commercial practice. These thermal indices are good predictors of thermal comfort at the herd or group level but with limited application at the individual animal level^22^. Current sensor systems can monitor behaviour, health and welfare aspects of individual cattle^38–42^ and accelerometer-based sensors^43–45^ show promise for monitoring cattle responses under intensive and extensive farming conditions. Accelerometer-based neck^16^ and ear-tag^22^ sensors can monitor cattle heat stress as determined by observed “heavy breathing” or “panting”. As such, these ear tag sensors could be deployed to infer the dynamic nature of cattle CBT under diverse thermal conditions.

Cattle response to heat varies depending in part on the characteristic differences that they acquired in the evolutionary process of natural selection, as well as during the domestication process to produce different subspecies from their early ancestors^46–49^. Humped/zebu cattle (*Bos indicus*) are recognised for their better heat-tolerance compared to non-humped/taurine cattle (*Bos taurus*)^50–52^, which is attributed to lower metabolic rate, larger sweat glands, less tissue resistance in heat flow from the body core to surface, paler coat and greater heat loss ability of zebu cattle^48^. Breed and coat colour influence heat stress response, which differs significantly between and within zebu, taurine and mixed-bred cattle^10,32,50–54^. Recent research has established that, in addition to breed and coat colour differences, there is substantial variation between individuals within the same breed and coat category in heat stress responses, and genomic selection can increase heat tolerance in cattle^22,55^. However, there is a paucity of work assessing the link between panting response during heatwave events and internal thermal conditions, tolerance or susceptibility to thermal stress. The causal pathways relating the thermal environment to cattle CBT and panting is not yet clearly understood.

In this work, we have continuously recorded individual panting data from 99 feedlot heifers using accelerometer-based ear tag sensors during summer for 77 days. Climatic data for the same period was collected from on-site weather station to calculate THI and HLI. Individual cattle panting score was visually recorded for two different heat wave events (during first 40 days) to identify heat-tolerant and heat-susceptible cattle. Selected heat-tolerant and heat-susceptible cattle were inserted with vaginal temperature data loggers during a third heat wave (day 63 to 66) to continuously record CBT. The association of CBT with simultaneously collected THI, HLI and panting data was explored with raw data and after removing diurnal pattern and long-term trend from all four time-series to investigate: 1) the differences in CBT of heat-tolerant and heat-susceptible cattle across differing level of THI and HLI, and 2) the suitability of automated panting data to detect heat-tolerant and heat-susceptible cattle compared with the individual cattle CBT and visual PS.

## Results

### Core body temperature and panting follow diurnal ambient thermal patterns and there is variation between cattle across hours of the day

**Figure 1:**
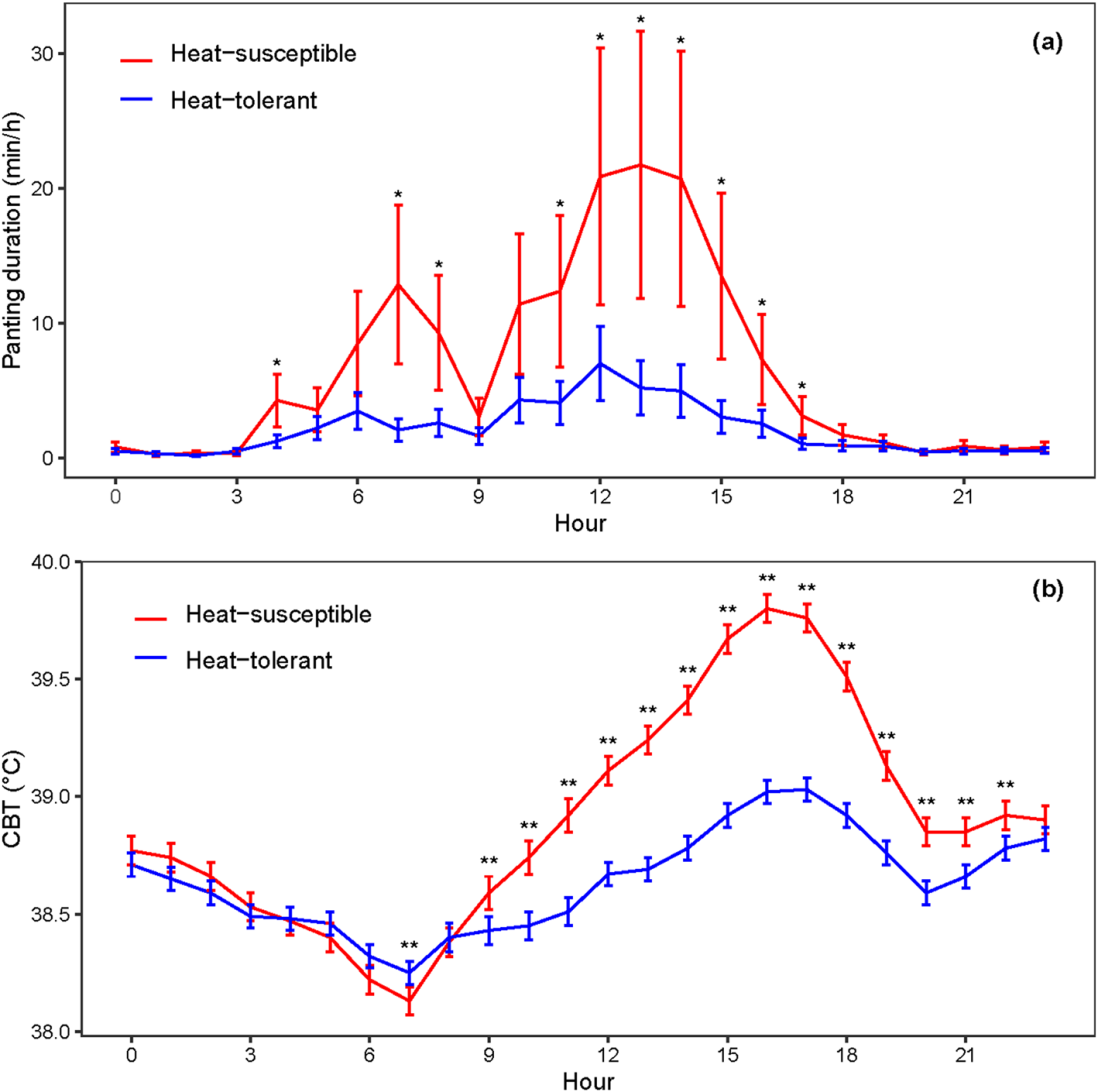
Differences in (**a**) panting duration and (**b**) mean core body temperature (CBT) between heat-tolerant and heat-susceptible cattle across hours of the day based on raw data. Average visual panting score (PS) data of first 40 days on feed were used for categorising cattle into heat-tolerant (PS < 1, *n* = 11) and heat-susceptible (PS ≥ 1, *n* = 8). The automated continuous CBT and panting data were collected from day 63 to 66. Each cow had 410 (10-min time intervals) CBT and 68 (1-h time interval) panting duration records across 68.2 h of continuous observation. Asterisks denote when differences were statistically significant (* for *P* < 0.05, ** for *P* < 0.01) and the error bars denote standard errors (SE).

**Figure 2:**
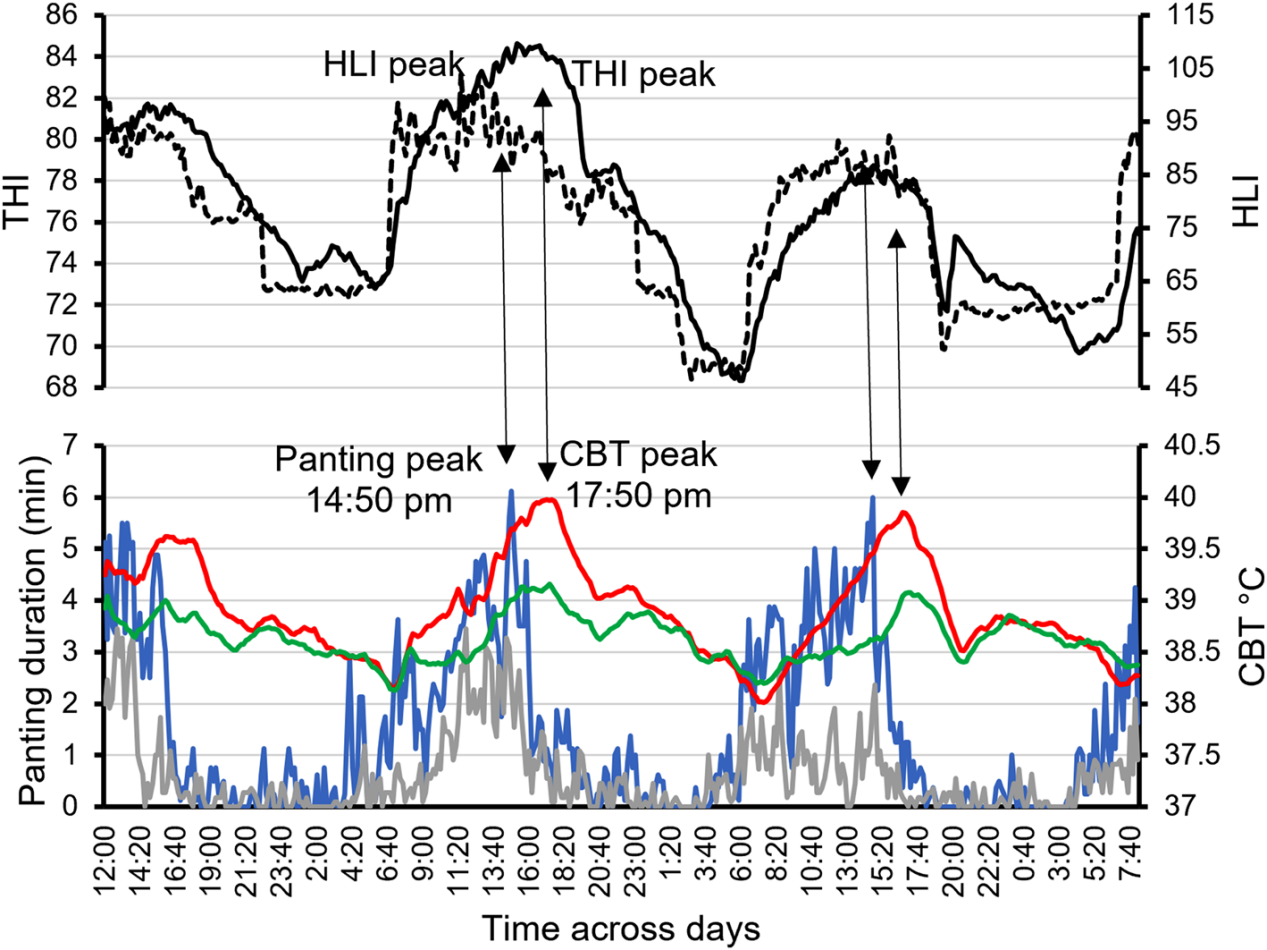
Association of temperature humidity index (THI) and heat load index (HLI) with core body temperature (CBT) and panting duration using raw data averaged at 10-min intervals. The solid and broken black lines in the upper panel represent THI and HLI, respectively. In the lower panel, red and green lines indicate CBT of heat-susceptible and heat-tolerant cattle, respectively, and the blue and grey lines indicate panting duration of heat-susceptible and heat-tolerant cattle, respectively. Vertical arrows indicate the connection of peak panting and CBT to peak HLI and THI, respectively.

Figure 1 shows the variation in CBT and panting response in cattle across hours of the day. Mean CBT and panting duration based on raw data differed between heat-tolerant and heat-susceptible cattle across the hotter part of the day. A rise in panting occurred 2–3 h earlier than the CBT rise and panting almost subsided at around 17:00 when CBT was at its peak. The 10-min interval time-series data suggest a close association of panting to HLI, and CBT to THI with distinct lags and a common diurnal pattern between these series (Fig. 2). The average CBT for the heat-susceptible and heat-tolerant heifers were 38.91 °C and 38.64 °C, respectively. Maximum CBT occurred around 17:20 (means: heat-susceptible: 39.76 °C; heat-tolerant: 39.00 °C), while minimum CBT occurred around 07:10 (means: heat-susceptible: 38.24 °C; heat-tolerant: 38.13 °C). The average panting for the heat-susceptible heifers was 1.56-min/10-min (logit(Pant) = − 1.62) compared with 0.63-min/10-min (logit(Pant) = − 2.05) for the heat-tolerant heifers. Peak panting occurred at around 13:00 (22 ± 10 min/h) for heat-susceptible, compared with at around 12:00 (5 ± 2 min/h) for heat-tolerant animals, while no/minimal panting occurred between 19:00 and 04:00 for both heat susceptible and heat-tolerant animals (Fig. 1). Time-series plots for THI (Supplementary Fig. S6) and HLI (Supplementary Fig. S7), similarly decomposed into their components (trend, 24-h cycle and residual) show that peak THI and HLI are around 14:40 and 11:30, respectively, while the corresponding minima are around 05:50 and 03:50, respectively. Raw values of THI and HLI are positively correlated (*r* = 0.83), although their residuals less so following decomposition (*r* = 0.51).

### Sensitivity of CBT to changing thermal environment depends on stress levels and cattle types

**Figure 3:**
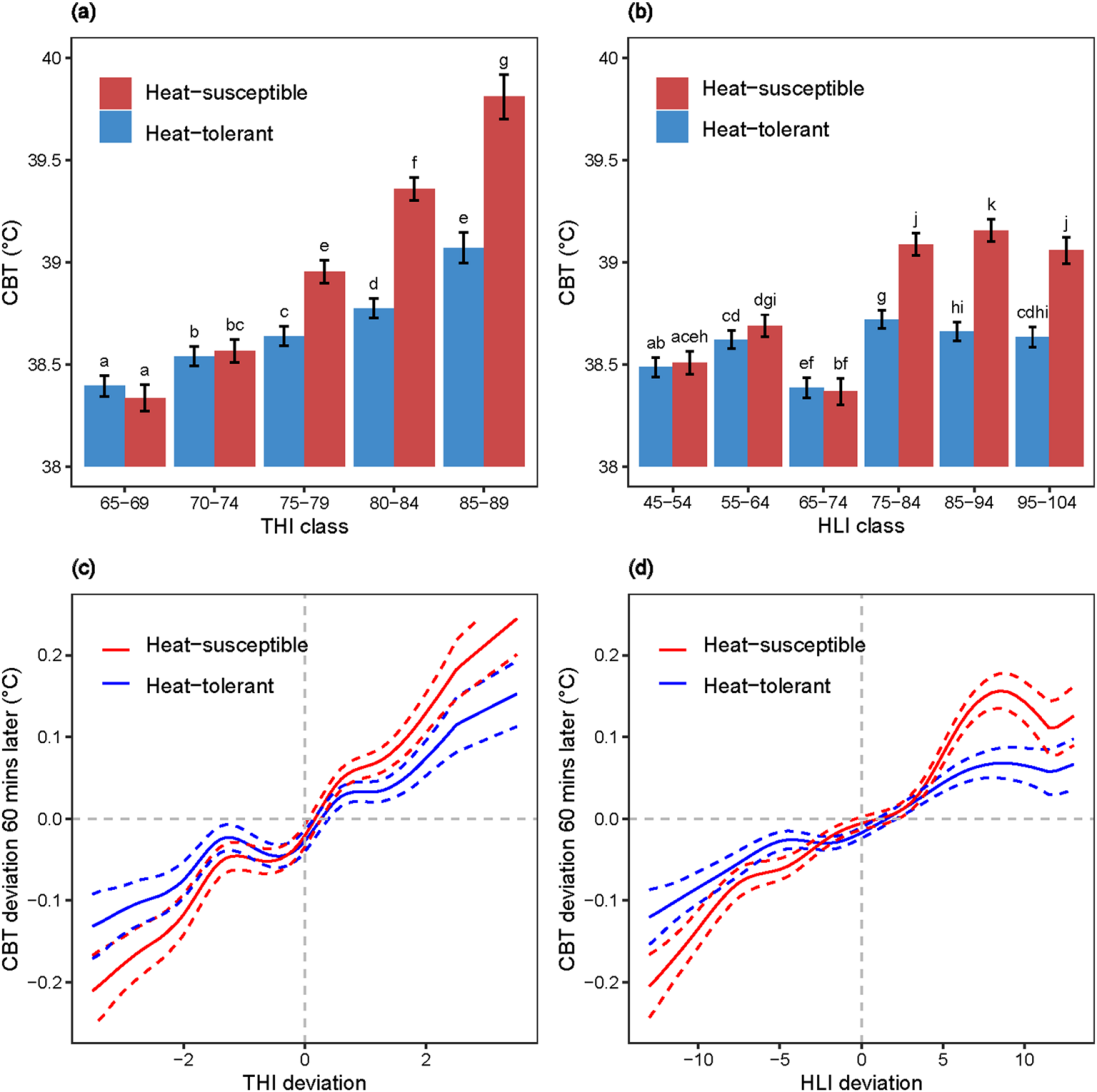
Core body temperature (CBT) response to different THI/HLI classes (based on raw data) (**a**) and (**b**) and to THI/HLI deviations (based on decomposed time-series data) (**c**) and (**d**) for heat-tolerant and heat-susceptible cattle. Error bars in (**a**) and (**b**) indicate standard errors of mean (SEM) and the means with different letters differ significantly. The dotted lines in (**c**) and (**d**) indicate standard errors of the CBT response curves as determined from decomposed CBT and THI/HLI time-series data.

Raw CBT, THI and HLI data-based results suggest that CBT increases with increasing THI and HLI values (Fig. 3), except for a slight depression of CBT at the HLI range of 65 to 74 within our data set under the conditions specified for this experiment. However, CBT response differed between cattle types after a THI range of 70 to 74 and a HLI range of 65 to 74 (Fig. 3a and b). Cross correlation analyses (Supplementary Fig. S1) with raw data suggest CBT lagged THI (*r* = 0.52) and HLI (*r* = 0.49) by 80 and 190 min, respectively. However, decomposed data suggest a 1-h lagged CBT response for both THI (*r* = 0.27) and HLI (*r* = 0.30).

Using these lagged time-series, mixed models were used to assess the associations more formally, particularly to assess differences between the heat-tolerant vs heat-susceptible animals and also to examine if the response to varying THI and HLI was linear or nonlinear (Fig. 3c and d). Since the analysis was conducted on the residual time-series, the approach here was to examine how a *change/deviation* in THI or HLI result in a *change/deviation* in CBT 60-min later, i.e. not to quantify the actual THI-CBT or HLI-CBT relationships. It can be seen that there was a lot of variation in responses between individual heifers to both varying THI and HLI; while some show little change, others show marked increases with increases in both of these thermal indices (Supplementary Fig. S2). However, what is apparent, particularly from the plot of the means curves is that the heat-susceptible heifers show a greater response to an increase in either index value, compared with heat-tolerant animals (Fig. 3c and d). These are indicated by the linear regression coefficients (heat-susceptible vs heat-tolerant: 0.0620 vs. 0.0376 for THI and 0.0136 vs. 0.0075 for HLI) from the fitted model, with the differences in regression slopes for HLI being significant (*P* = 0.032), but marginal for THI (*P* = 0.055).

**Figure 4:**
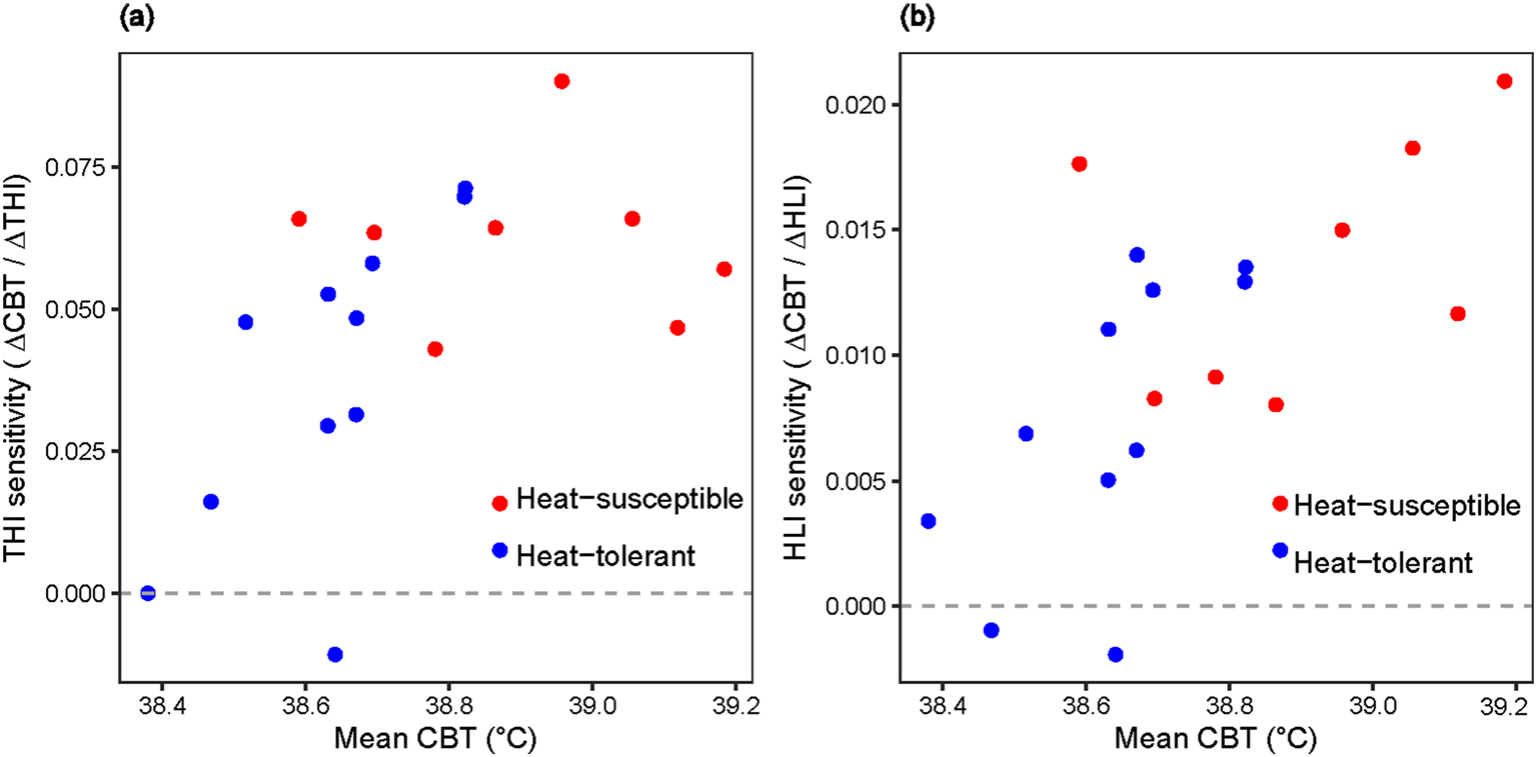
Sensitivity of core body temperature (CBT) change on THI and HLI deviations. Slopes of the individual animal CBT response curves across THI and HLI deviations were plotted against the average recorded CBT of each animal.

Given that spurious associations occur with raw time-series, emphasis should be placed in the residual time-series obtained from the decomposed data, and from this it is noted that CBT has a maximum association with both THI and HLI 60-min later. These residual time series data also revealed that there are thermal zones where CBT was similar in both cattle types, and beyond that zone heat-susceptible cattle showed greater sensitivity to THI and HLI deviations (Fig. 4a and b) for CBT change. In cooler THI/HLI conditions, heat-susceptible cattle maintained a lower CBT than heat-tolerant cattle, that is they dropped their CBT most through the release of body temperature in the cooler periods. However, the opposite is seen when thermal load increases, the CBT of heat-susceptible cattle rose significantly more than that of heat-tolerant cattle (Fig. 3c and d).

### Panting response in cattle is associated more closely with thermal environment than to CBT

**Figure 5:**
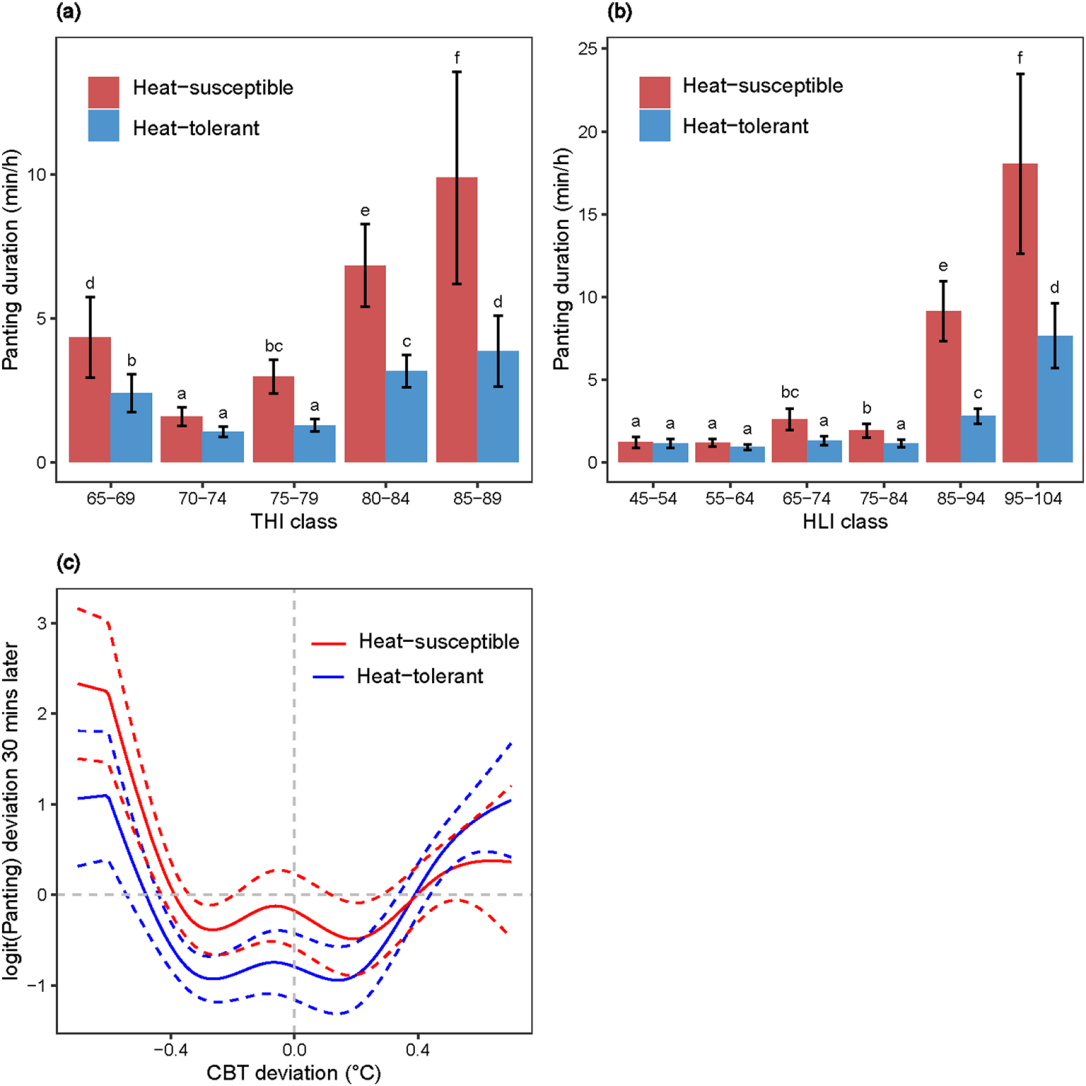
Panting response to different THI/HLI classes (based on raw data) (**a**) and (**b**) and to CBT deviations (based on decomposed time-series data) (**c**) for heat-tolerant and heat-susceptible cattle. Error bars in (**a**) and (**b**) indicate standard errors of mean (SEM) and the means with different letters differ significantly. The dotted lines in (**c**) indicates 95% confidence intervals of the panting response curves as determined from decomposed CBT and panting time-series data. Logit function was used as panting was recorded at minute level as a binary (Yes = 1, No = 0) outcome.

Hourly panting duration corresponding to each THI and HLI class based on raw data suggests that heat-susceptible cattle spent comparatively greater time panting than heat-tolerant animals across THI and HLI categories (Fig. 5a and b). Figure 5 shows that panting responses were lowest and similar for both cattle types at a THI range of 70–74 instead of at the lowest recorded THI range of 65–69, and HLI range of 45–64 under our experimental conditions. Using the raw data, the cross-correlation of panting with lagged CBT shows a greater association at a back-lag of twelve 10-min intervals (*r* = 0.39), i.e. CBT responds to panting about 120-min later. When looking at the cross-correlation of the residual components, earliest maximum positive association was found with a 30-min forward-lag (*r* = 0.06) (Supplementary Fig. S3). Cross correlation analyses with raw panting and thermal data revealed that thermal condition impacts panting with a comparatively smaller back-lag (80 min) than that of CBT; residual components revealed immediate maximum positive association with no lag (Supplementary Fig. S3). Mixed model-based estimates with decomposed data suggest panting deviations of both cattle types are similar for the same level of deviations in CBT (Fig. 5c). As noted above, emphasis should be placed on the residual time-series, and it is noted that panting is distantly associated with CBT.

## Discussion

This research is the first to continuously record animal thermal response along with thermal indices (THI and HLI) and reveal CBT and panting levels for heat-susceptible and heat-tolerant cattle. From the raw data, a clear 24-h cycle is present with some evidence of higher CBT and panting for heat-susceptible (high-panting) heifers. This differential is particularly evident when looking at the decomposed longer-term trends in CBT and panting (Supplementary Figs. S5 and S8), with the average CBT for the heat-susceptible heifers being 38.91 °C compared with 38.64 °C for the heat-tolerant (low-panting) heifers. Peak panting preceded peak CBT by 2 to 3 h with a close association to peak HLI. In contrast, peak THI was closely associated with peak CBT with a lag of 80 min. However, the decomposed data revealed that CBT has a 1 h lag response to both THI and HLI. Both raw and decomposed data revealed that CBT and panting duration for heat-susceptible cattle are more sensitive to changes in thermal indices compared with heat-tolerant cattle.

The association of CBT, thermal indices and panting across time with raw data can be misleading due to the common diurnal and seasonal pattern in these time-series^18,56^ and the potential biological lag between cause and effect^11^. Also, individual variation^22,51,57^ in heat-tolerance and heat-susceptibility may impact these associations. For example, there was a decline in CBT from a lower HLI class (55–64) to higher HLI class (65–74). This was due to the transition in HLI occurring in the cooler hours of the day whilst cattle continued to dissipate body heat due to the lagged response to ambient thermal conditions. For the same reason, panting duration was reduced at a THI class of between 70 to 74. Similarly, differential animal responses (panting score and CBT) have been recorded at similar thermal index values for morning and evening observations^29^. Decomposing these time-series into residual components^58^, assessing them with a cross-correlation function, and allowing for individual variation in analyses may help to establish causal relationships and avoid spurious cross-correlation between series. Decomposed data revealed that both THI (*r* = 0.27) and HLI (*r* = 0.30) impact CBT with a forward lag of 1 h, showing the ability of cattle to maintain CBT at a set range independent of immediate thermal conditions^50^. A greater lag of 4 to 5 h has been indicated in previous research but was based on raw data^11,12^. However, after removing diurnal and long-term trend patterns from raw THI and CBT data, a lag of 2 h in dairy cattle has been reported for THI greater than 72^18^. Our research was conducted using feedlot heifers during heat wave events with greater thermal load (maximum range: THI 81 to 84 and HLI 95 to 100), which may have reduced the lag between thermal conditions and CBT. With the same lag (1 h) as THI, HLI had a greater association with CBT as expected as HLI includes more ambient thermal factors (e.g. solar radiation)^23^ than THI. To our knowledge there is no other research exploring the association between HLI and CBT after decomposing time-series data. Both heat-tolerant and heat-susceptible categories of cattle were able to maintain similar CBT for smaller deviations in THI/HLI, indicating a thermoneutral zone. However, deviations were significantly greater for the heat-susceptible cattle beyond that zone. This explains the differential thermoregulatory ability of cattle beyond the thermoneutral THI (68–72)^59^ and HLI (70–77)^23^ values. Thus, it can be inferred that cattle that can dissipate body heat effectively at thermal conditions with greater THI/HLI due to superior thermoregulatory ability are heat-tolerant, and those that cannot do so, are heat-susceptible.

The deviations in panting duration in response to deviations in CBT were similar for heat-tolerant and heat-susceptible cattle categories. This finding suggests that the basic biological and thermoregulatory association between CBT and panting is maintained across cattle categories. Similarly, numeric panting scores assessed during morning, mid-day and afternoon observations were associated with CBT thresholds in a time-dependant manner^29^. However, there is no research with continuous panting duration data to compare with our results. At a particular CBT, both heat-tolerant and heat-susceptible cattle will have a similar panting response. However, differences arise due to a comparatively greater deviation in CBT for heat-susceptible cattle in response to similar deviations in THI/HLI. As such, heat-susceptible cattle will have a greater CBT under similar thermal stress conditions. Therefore, if compared simultaneously, heat-susceptible cattle will exhibit a greater CBT deviation at similar thermal stress conditions and corresponding greater durations of panting when compared with heat-tolerant cattle. Decomposed long-term trend data revealed heat-susceptible cattle to maintain overall greater mean panting durations and CBT compared with heat-tolerant cattle across THI and HLI values during the experimental days. The heat-tolerant and heat-susceptible animals were identified based on the average panting score of individual cattle during two consecutive heat wave events prior to the automated CBT data recording during a third heat wave event. Therefore, our sensor-derived panting data can be used as a reliable and repeatable indicator of heat-tolerance and heat-susceptibility of cattle as determined by individual mean panting scores.

According to the extended local equilibria rules of thermodynamics^60^, different segments of an object may have differential heat exchange towards a balance between heat loss and heat gain to and from the surrounding environment to maintain thermal homeostasis. Along with this basic principle, the biological thermoregulatory ability of cattle (being homeothermic) helps with maintaining the CBT within a set limit of 38–39 °C^6,7^ under a range of ambient conditions (5–25 °C)^8^ usually by ‘sensible’ (conduction, convection and irradiation)^50,61^ heat exchange between an animal’s body and its surrounding environment. Ambient thermal load beyond the upper critical limit (25 °C) of thermoregulatory ability causes an increase in CBT followed by activation of ‘insensible’ heat dissipation mechanisms (sweating and panting)^48,51^. Based on this, one possible causal pathway is:

1 $${\text{THI/HLI}}\, \to \,{\text{CBT}}\, \to \,{\text{Panting}}{.}$$

The first segment of this path (THI/HLI → CBT) is supported by the finding that ambient thermal conditions have an impact on cattle CBT 1 h later. In addition to the linear trend of increasing CBT with increasing THI and HLI, there was strong evidence for a nonlinear trend, as indicated in our results (Fig. 3, both *P* < 0.0001). For both THI and HLI, there are zones in the middle ranges where changes do not greatly affect CBT, perhaps indicating a ‘homeostasis effect’. But beyond that, there are more substantial changes in CBT. Similarly, the CBT of dairy cattle was not affected by THI of less than 72^18^. Therefore, CBT may not represent the immediate thermal stress level of cattle. However, an evaluation of the second segment (CBT → Panting) failed to indicate an association of CBT with panting. Consequently, a different cause-effect pathway was also hypothesised: 2

The analyses of the “THI/HLI → Panting” pathway did not result in either a significant correlation or a meaningful association between the deviations in thermal indices and the deviations in panting. Therefore, the association between CBT and panting is not straight forward, as it seems they both impact each other positively and negatively with variable lag via some other factors or phenomena. Intrinsically, panting of cattle can be controlled by complex homeostatic and heat dissipation mechanisms. The genetic and coat colour variation may impact individual panting behaviour^22–24^ primarily through differential sweating responses^9^ to hot conditions. Data on skin or surface temperature may be useful in further studies given the principle that panting is initiated by a parasympathetic nervous response^16^ sensitive to the skin surface temperature. While our approach throughout this experiment was mostly observational, a controlled experimental approach is needed to unveil the complex relationship between CBT, panting and thermal indices in the context of other heat loss effector pathways^62^ such as conduction, convection, radiation, and evaporation (breathing, sweating, etc.).

## Methods

This research was conducted at a commercial feedlot in south-eastern Queensland, Australia, from December 2019 to February 2020, as approved by The University of Sydney Animal Ethics Committee (Approval No. 2017/1213). All methods were performed in accordance with the guidelines and regulations of the Ethics Committee and is reported in accordance with ARRIVE guidelines.

### Animals and feedlot induction

**Figure 6:**
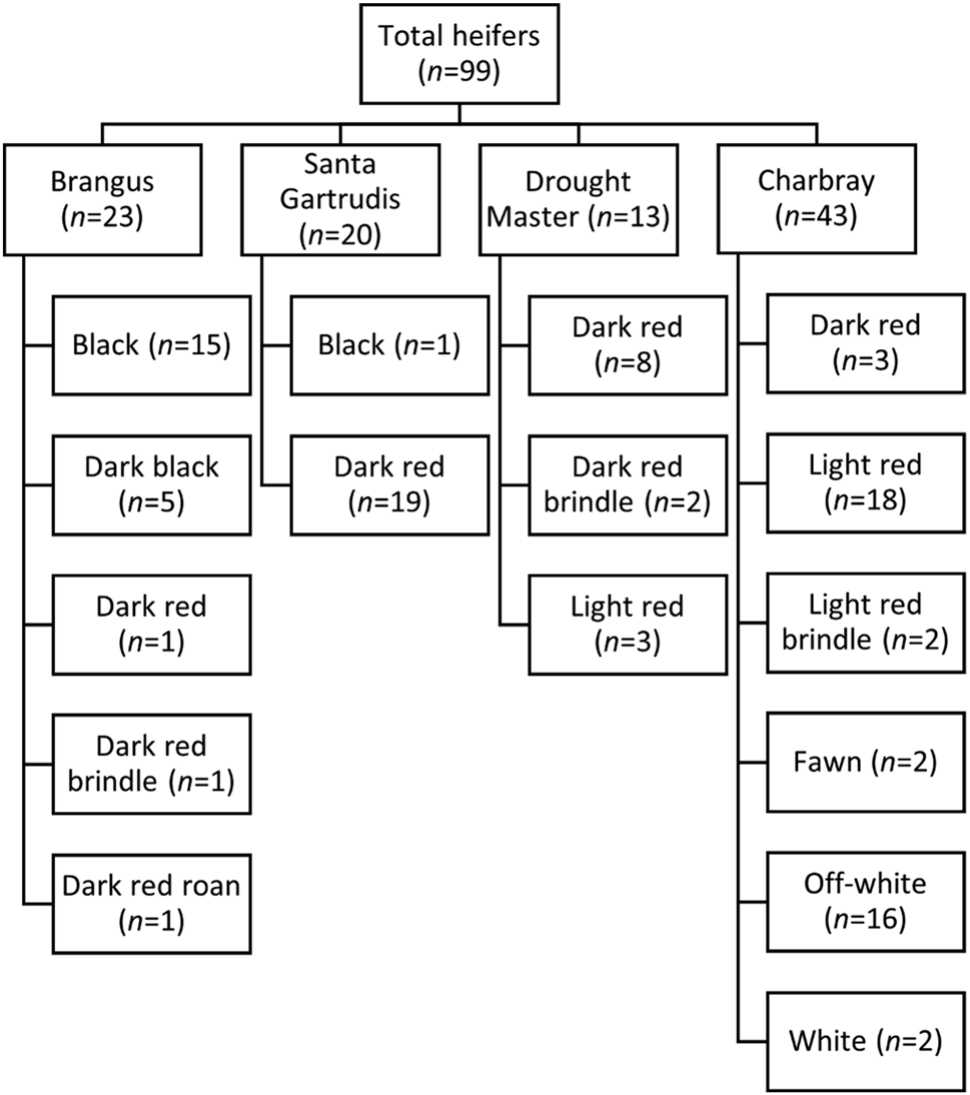
Breeds and coat colour categories of cattle used in this experiment.

A pen of 99 beef heifers consisting of four colour categories namely white (*n* = 18) including white and off-white coat; light red (*n* = 23) including light red, light red brindle and fawn coat; dark red (*n* = 35): including dark red, dark red brindle and dark red roan coat; and black (*n* = 21) including black and dark black coat, and mixed-breed categories consisting of four breeds namely Charbray, Drought Master, Santa Gertrudis and Brangus were used for this experiment. A hierarchical scheme of animal categories is presented in Fig. 6. The recorded mean live weight of cattle at induction was 339 ± 1.4 kg (mean ± SE) at an age of approximately 2 years old based on dentition score. Cattle were vaccinated against respiratory disease caused by *Mannheimia haemolytica* (Bovilis MH, Coopers Animal Health, NSW, Australia) and Infectious Bovine Rhinotracheitis (IBR) (Rhinogard, Zoetis Animal Health, New Jersey, USA), and had an application of anti-parasitic pour-on backline (Fasimec™ Cattle Pour-On (triclabendazole, abamectin), Ellanco Australasia, NSW, Australia). A single-piece self-piercing visual ID tag (Allflex^®^ Livestock Intelligence™) and a behaviour-monitoring sensor (Allflex^®^Livestock Intelligence ™ eSense™Flex, SCR Engineers Ltd., Netanya, Israel) were fitted in the outer and central part, respectively, of the left ear since the national livestock identification system (NLIS) tag was located in the right ear. Sensors were placed by trained staff as per the manufacturer’s installation guideline^63^.

### Feed and feeding schedule

The animals were offered ad-libitum feed and habituated to a barley-based finisher diet through three transitional diets over a period of 14 days. The final finisher diet was composed of 47% barley grain, 20% biscuit meal, 17% barley silage, 6% canola meal, 5% liquid finisher supplement for feedlot cattle (Performance Feeds, Brandon, QLD 4808, Australia), 3% cotton seed oil, and 2% sorghum hay. The daily allowance of the finisher diet was offered in portions of 20, 20 and 60% at 08:00, 10:00 and 13:00, respectively, from day 15 to the end of the feed period.

### Climate data, thermal indices and heat wave events monitoring

**Figure 7:**
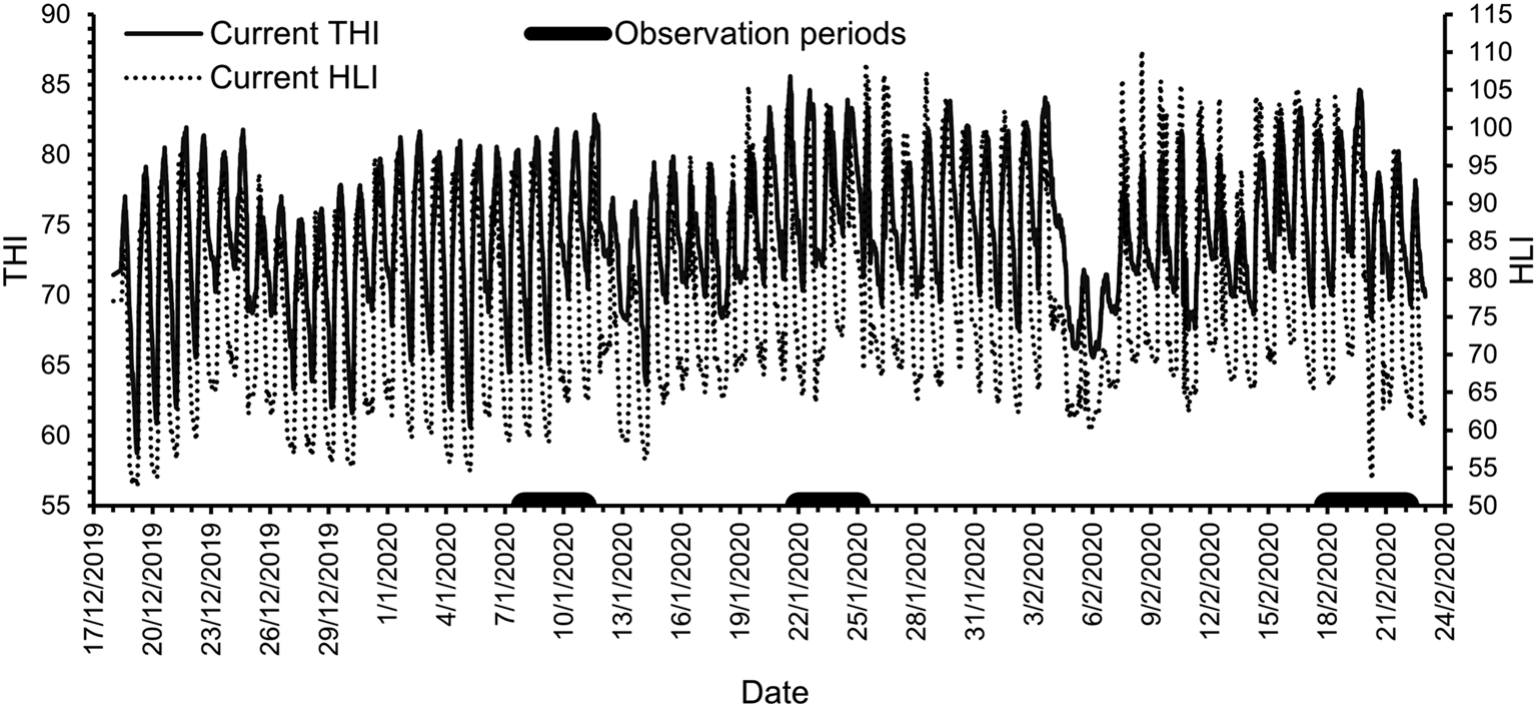
Temperature humidity index (THI, solid line) and heat load index (HLI, dotted line) calculated at 10-min intervals across the experimental days. The solid bars on the *x*-axis indicate the three observation periods. Data were recorded every 10-min across 24-h.

Climatic data (dry bulb air temperature, black globe temperature, relative humidity, solar radiation, wind speed) were collected at 10-min intervals from an on-site weather station. These data were used to calculate THI as per the equation used by Islam et al.^22^, and HLI according to Gaughan et al.^23^ across the experimental period including three consecutive heat wave events (days 22–24, 36–38, and 63–66 on feed) when panting scores were recorded (Fig. 7). The automated CBT monitoring occurred only during the third heat wave event. The average minimum, mean and maximum THI values were 66, 75 and 82; 72, 78 and 84; and 70, 76 and 81 for heat wave period 1, 2 and 3, respectively. The corresponding values for HLI were 54, 74 and 96; 58, 79 and 100; and 56, 76 and 100.

### Panting monitoring

Individual cattle behaviour and activity were automatically recorded from day 1 to the end of the experiment (day 77) and sent wirelessly to a base station by the ear-attached behaviour monitoring sensors (Allflex® Livestock Intelligence ™ eSense™Flex, SCR Engineers Ltd., Netanya, Israel). The animal response recorded in each minute was classified into “heavy breathing (panting)” or “a variety of behavioural states other than panting (not panting)” according to a proprietary algorithm (Heatime^®^ Pro + software from Allflex^®^ Livestock Intelligence™; SCR Engineers Ltd., Netanya, Israel). Although the proprietary algorithm used for this work has been optimised for mature taurus dairy cattle, we have previously validated these monitoring sensors for the detection of panting during moderate heat wave periods^22^.

A general health and welfare check (respiration, gait, and demeanour) of animals was performed by trained staff twice daily as per the routine feedlot protocol. Visual recording of panting was performed by an experienced observer (the first author) between 08:00 and 17:00 during selected heat event periods based on weather forecast. Panting scores were assigned as per the guideline described by Islam et al.^22^ (Table 1). Observation was continuous during the aforementioned time period, however, scoring was opportunistically performed by instantaneous scan only when an individual is identified panting with higher intensity. Individual animal records were maintained by recording visual ID number, and binoculars were used to read the number for a distant animal. Animal observations were performed maintaining at least 10 m distance from the fence line, feed pen and water trough to minimise disturbance of the cattle.

**Table 1:** The respiratory dynamic and panting score (PS) of cattle.

PS	Description
0	Normal breathing, no forward–backward heaving
1	Forward–backward heaving, mouth closed, no drool or foam, easy to see chest movement
2	Forward–backward heaving, mouth closed, but drool or foam present
3	Forward–backward heaving, mouth open or intermittent mouth open, excessive drooling, tongue not extended, neck extended, and head held up
4	Forward–backward heaving, open mouth with tongue protruding either occasionally or for prolonged periods, excessive drooling, neck extended, head held up or down

### Identification of heat-tolerant and heat-susceptible cattle

Heat-tolerant and heat-susceptible cattle were identified based on the aforementioned visual monitoring of panting across the day during early- and mid-feed periods, respectively from day 22–24 and 36–38 in feed. When an animal was found panting at any intensity from panting score 1–4, an instantaneous scan was performed simultaneously to find animals with no panting (panting score 0) or panting at a lower intensity than what is observed in the high-panting animal. Animals were ranked based on their average panting score across the observation days. Subsequently for core body temperature monitoring, we classified the 13 most high-panting individuals as “heat-susceptible” (average PS ≥ 1) and the 13 most no/low-panting individuals as “heat-tolerant” (average PS < 1).

### Monitoring core body temperature (CBT)

A pilot study was conducted in November 2019 with the approval from The University of Sydney Animal Ethics Committee (Approval No. 2019/1659) to evaluate the in-vivo functionality of internal temperature logging device at The University of Sydney dairy facility (“May Farm”) in Camden, New South Wales, Australia. Briefly, we have tested vacuum sealed “thermocron iButtons” (*n* = 6, Code: DS1922L-F50, Resolution: 0.063 °C, Accuracy: 0.5 °C, Range: − 10 °C to + 65 °C; Maxim Integrated, San Jose, CA, USA) in a water bath at fluctuating temperatures from 30 to 50 °C. Then we prepared intravaginal temperature logging device (Supplementary Fig. S4) by attaching iButtons (water protected by sealing with thin polyethylene wrap and securing inside fingers of nitrile gloves with knot) to controlled internal drug release (CDR) devices with the help of heat-shrink tubing. The constructed device was validated in-vitro under the same water bath conditions and then tested in-vivo in dry cows under pastoral condition. The accuracy of the device was within the manufacturer’s recommendation. In-vivo testing revealed the device is capable of recording changes in CBT in response to ambient thermal conditions along with circadian rhythm.

For the present research, 20 more temperature loggers were tested (after waterproofing as per the pilot study) in a water bath setup to incorporate into the temperature logging CIDR devices. All temperature loggers were found to be within the range of manufacturer’s given accuracy. We used these 20 new and 6 previously used temperature loggers from the pilot study for the construction of internal temperature logging device. The loggers were programmed to record temperature at 1-min interval with 11-bit mode and at a resolution of 0.063 °C. Finally, the temperature logging CIDR devices (*n* = 26) were inserted into the vaginal cavity of the selected heat-tolerant and heat-susceptible feedlot heifers using a CIDR applicator (Eazi-Breed™ CIDR^®^ applicator, Zoetis Animal Health, New Jersey, USA) following a standard operating procedure as provided by the manufacturer. The temperature logging CIDR devices were inserted on day 63 of the 77-day feed period between 08:00 and 09:00, loggers were programmed to record vaginal temperature from 12:00 at 1-min intervals. The temperature logger memory allowed data to be recorded continuously up to 08:15 of day 66 on feed. Panting scores of CIDR-inserted heifers were monitored between 09:00 and 17:00 for the abovementioned four days. Temperature data recording was also for four days and the CIDRs were removed 7 days after insertion to minimise recurring drafting stress. Leaving the CIDRs for 7 days conformed with veterinary recommendations without any risk of infection and general monitoring remained unchanged during this period.

### Data retrieval

The CBT data were read and exported at the minute level from the loggers, and animal numbers were aligned with the corresponding CIDR identification mark. Two of the CIDR devices were lost from the heat-susceptible category of animals, and at the data reading stage we found five loggers (three from heat-susceptible and two from heat-tolerant category) with an error message and no data were retrievable from them. This resulted in continuous CBT data from eight heat-susceptible and 11 heat-tolerant individuals for analysis. Automated panting, CBT and thermal indices data were collated with the matching of date, time, and animal number for further analysis.

### Statistical analyses

The association of temperature humidity index (THI) and heat load index (HLI) with core body temperature (CBT) and automatically-recorded panting across time was investigated in two categories of feedlot cattle, heat-tolerant animals (n = 11) and heat-susceptible animals (n = 8). The minute-level CBT and panting data were averaged on a 10-min basis for each animal from day 63 (on feed) at 12:00 ending on day 66 at 08:10, which resulted in 410 observations per animal. Corresponding to each of these observations was the THI and HLI for the same times.

To determine the associations between thermal indices (THI and HLI), CBT and panting, processing of all four time-series data (CBT, THI, HLI and Panting) was required. Each of these series had long-term trends as well as 24-h cycles, which would cause spurious associations between them, regardless of any underlying functional association. Hence it was necessary to analyse data as raw as well as after removing these patterns, and investigate associations between the remaining or ‘residual’ components, i.e. to assess how a short-term change in THI or HLI effects a change in CBT or panting.

The time-series for each of the heifers were processed separately. The ‘stl’ function in R^64^ was used to decompose the series into 24-h cycle, trend and residual, i.e. CBT_raw = CBT_24-h cycle + CBT_Trend + CBT_Residual. The same method was then applied to decompose the THI, HLI and the panting series into its components (Supplementary Figs. S5, S6, S7 and S8). However, prior to decomposition of the panting series, since a binomial modelling approach was used, an empirical logit transformation was made:

3 $${\text{logit}}\left( {{\text{Pant}}} \right)\, = \,{\text{log}}_{{\text{e}}} \left[ {\left( {{\text{NPant}} + {1}} \right)/\left( {{1}0 - {\text{NPant}} + {1}} \right)} \right],$$

where NPant is the number of minutes in a 10-min interval where panting was detected. The residual components for the four time-series were then carried forward for analysis and these are considered ‘stationary’, an essential step in time-series analysis^58^. Next, in order to assess any time lag between change in one time-series and change in another series, the cross correlation between the residual time-series was evaluated and plotted using the ‘ccf’ function in R.

Having assessed the most likely time lag between the thermal indices and response in CBT, and between the CBT and response in panting; linear mixed models were used to explore the associations in more details. Having some of the weather variables common in both the indices and because of the high correlation between THI and HLI, they were not considered as simultaneous predictors in the same model. Fixed effects for the model exploring the association of CBT and thermal indices were THI_Residual/HLI_Residual (covariate), cattle category (‘Heat-tolerant’ and ‘Heat-susceptible’) and their interaction, allowing for a different response slope for each cattle category. To allow for any nonlinearity in the response, smoothing splines were used for the effect of THI_Residual/HLI_Residual, allowing for an overall trend, trends for each cattle category, and trends for each animal within each cattle category. These spline terms were included as random effect terms in the model, along with an overall random effect for each heifer. A similar model including CBT_Residual (covariate), Cattle category (‘Heat-tolerant’ and ‘Heat-susceptible’) and their interaction as fixed effects along with similar smoothing splines for CBT_Residual were used to explore association between CBT and panting (logistic mixed model, number of minutes out of 10-min interval was classified as panting). Association of panting (response variable) with thermal indices (explanatory variables) were analysed in the same way as for CBT and thermal indices. Model fitting was conducted using ASReml-R Release 4 in the R environment^65^.

## Supplementary Information

Supplementary Figures.

## Data Availability

All data except the raw accelerometer-based sensor data (proprietary for the manufacturer) are available upon request from the corresponding author. Public sharing is not possible due to competing interest between manufacturers of the sensor technology we used.
