# Supplementary material for: Revealing the diversity of internal body temperature and panting response for feedlot cattle under environmental thermal stress
Source: Sci Rep. 2023 Mar 25;13:4879. doi: 10.1038/s41598-023-31801-7 (PMC10039931; doi:10.1038/s41598-023-31801-7)
Supplement: Supplementary file 1 — Supplementary Figures. [file 41598_2023_31801_MOESM1_ESM.pdf]

Appendix A

Revealing the diversity of internal body temperature and panting response for feedlot cattle under environmental thermal stress

M. A. Islam, S. Lomax, A. K. Doughty, M. R. Islam, P. C. Thomson, C. E. F. Clark

Supplementary figures

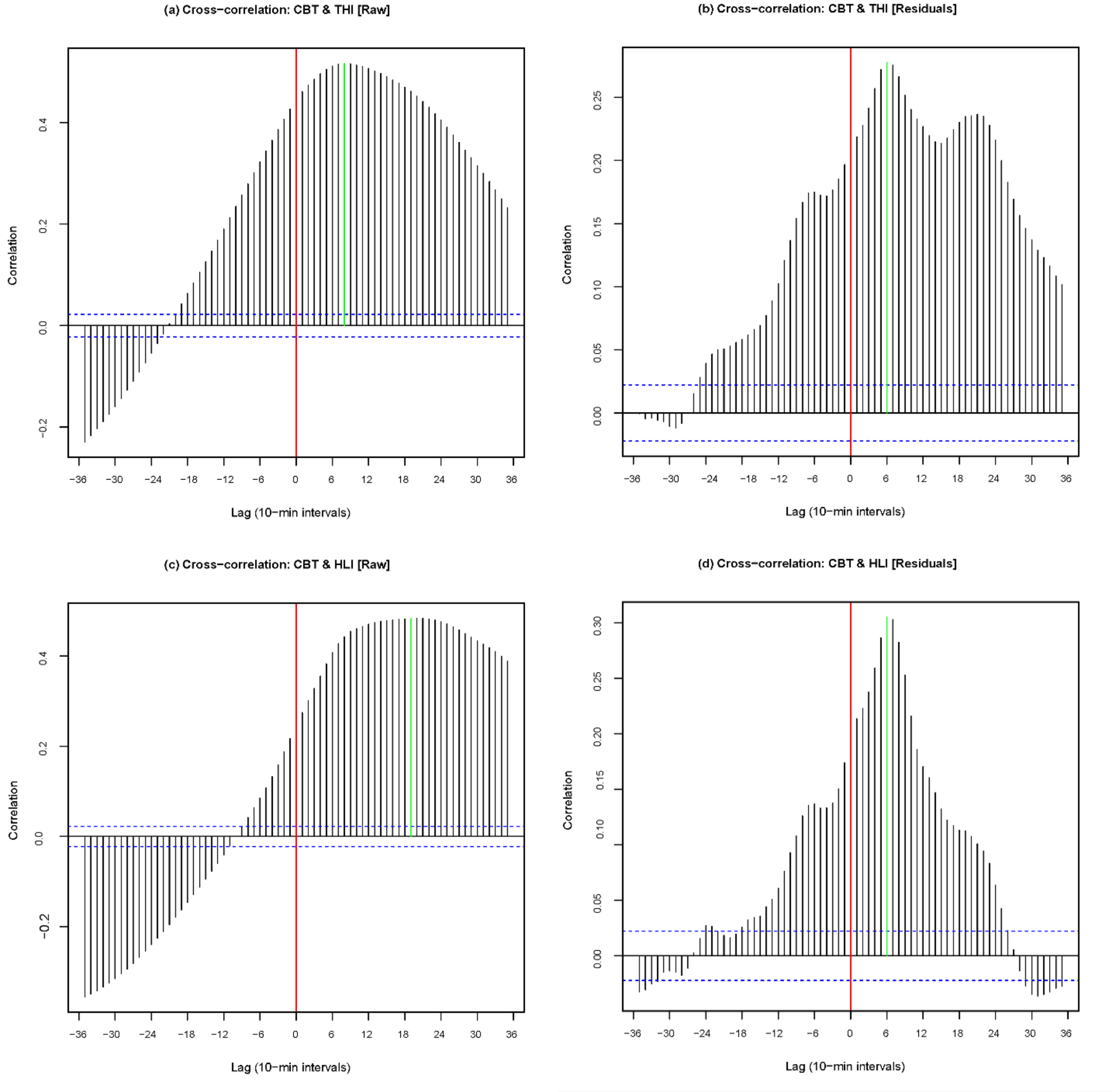

Supplementary Figure S1. Cross-correlation plots between CBT and the two thermal indices (THI and HLI) at different time lags (intervals of 10-min). The left panels are based on raw data and the right panels on decomposed residual deviations for each time-series. The red vertical line is drawn at a lag of 0-min (i.e., association at the same time point), the green vertical line is drawn at the lag at which maximum correlation occurs, and the blue horizontal lines are the threshold significance levels for the correlations.

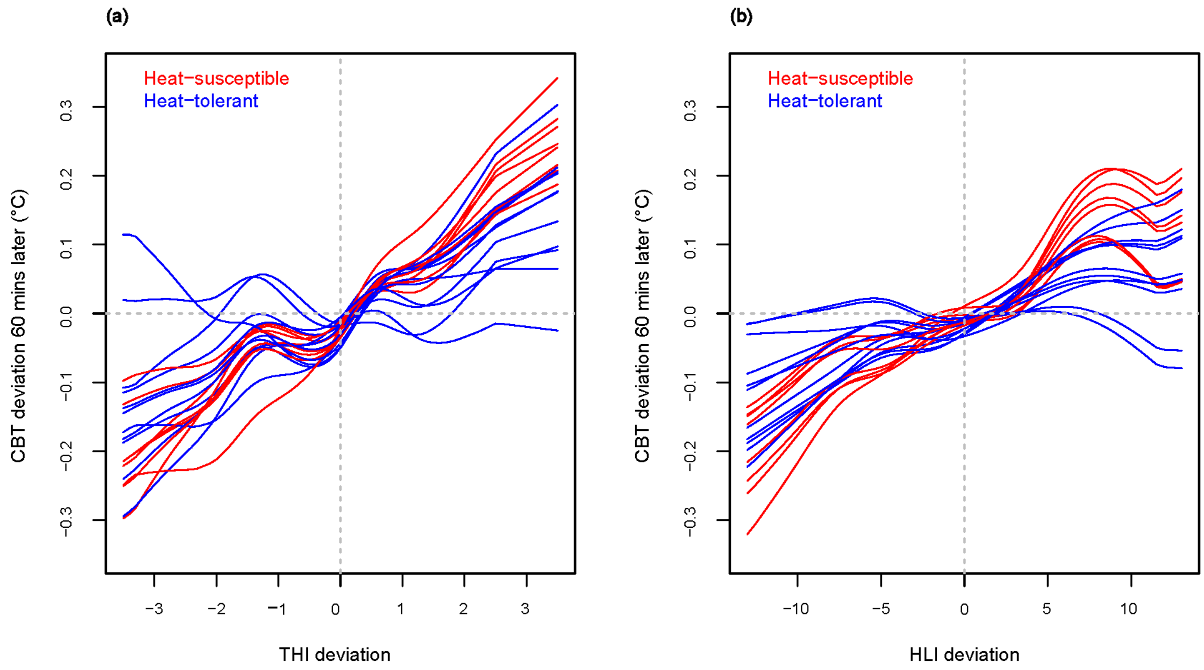

Supplementary Figure S2. Individual responses of CBT to varying THI and HLI: mixed model predictions based on a lagged response of 60-min. The heat-susceptible and heat-tolerant individuals are indicated by red and blue lines, respectively.

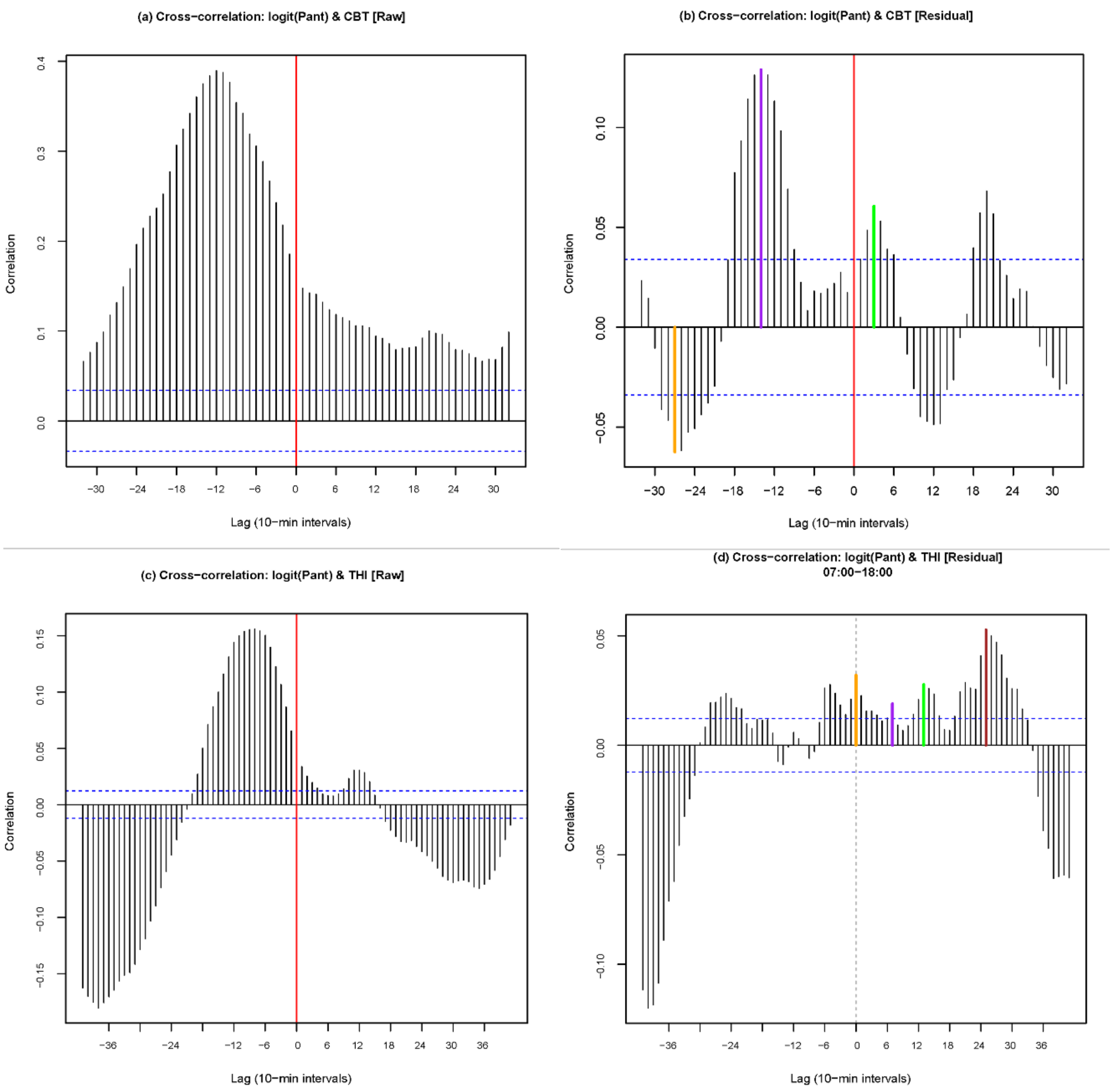

Supplementary Figure S3. Cross-correlation plots between core body temperature (CBT) and panting, and temperature humidity index (THI) and panting at different time lags (intervals of 10-min). Logit function was used as panting was recorded at minute level as a binary (Yes = 1 and No = 0) outcome. The left panels are based on raw data and the right panels on decomposed residual deviations for each time-series. The red vertical lines in the first three panels and the blue vertical line in the last panel are drawn at a lag of 0-min (i.e., association at the same time point). Peak correlation points with variable lags have been highlighted with different colours, and the blue horizontal lines are the threshold significance levels for the correlations.

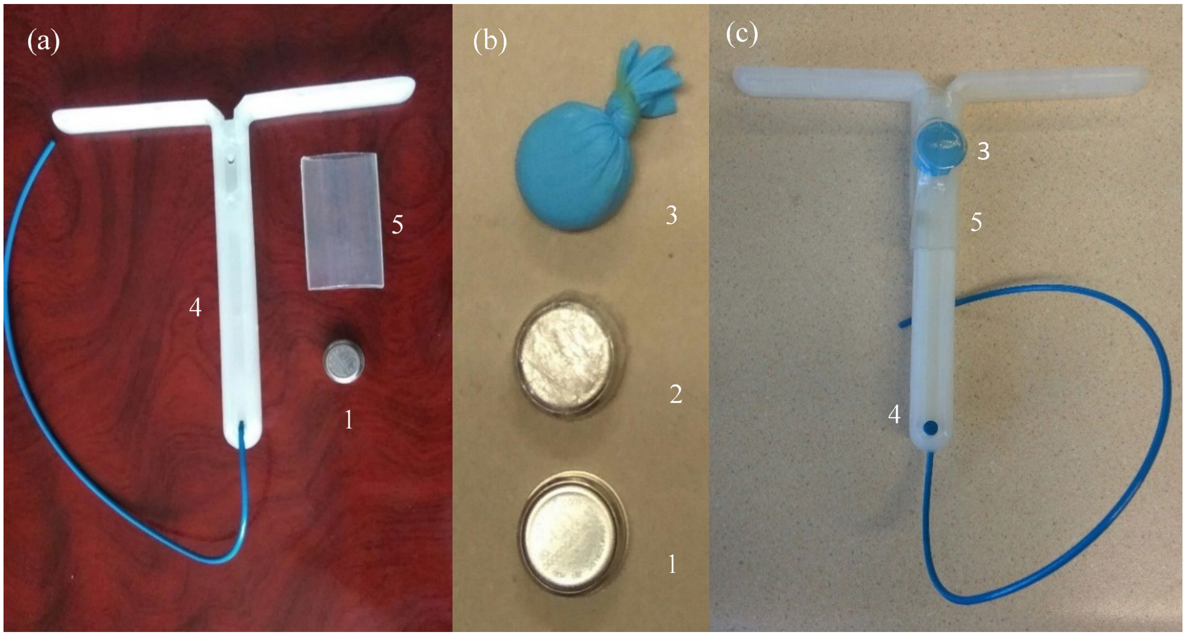

Supplementary Figure S4. Construction of indwelling (intravaginal) temperature logging system. (**a**) Basic requirements, (**b**) Water proofing of temperature logger (iButton) and (**c**) The final device; 1 = iButton, 2 = iButton wrapped with cling wrap, 3 = iButton secured within nitrile glove finger, 4 = empty/clean controlled internal drug release (CIDR) device, and 5 = transparent heat shrink tube.

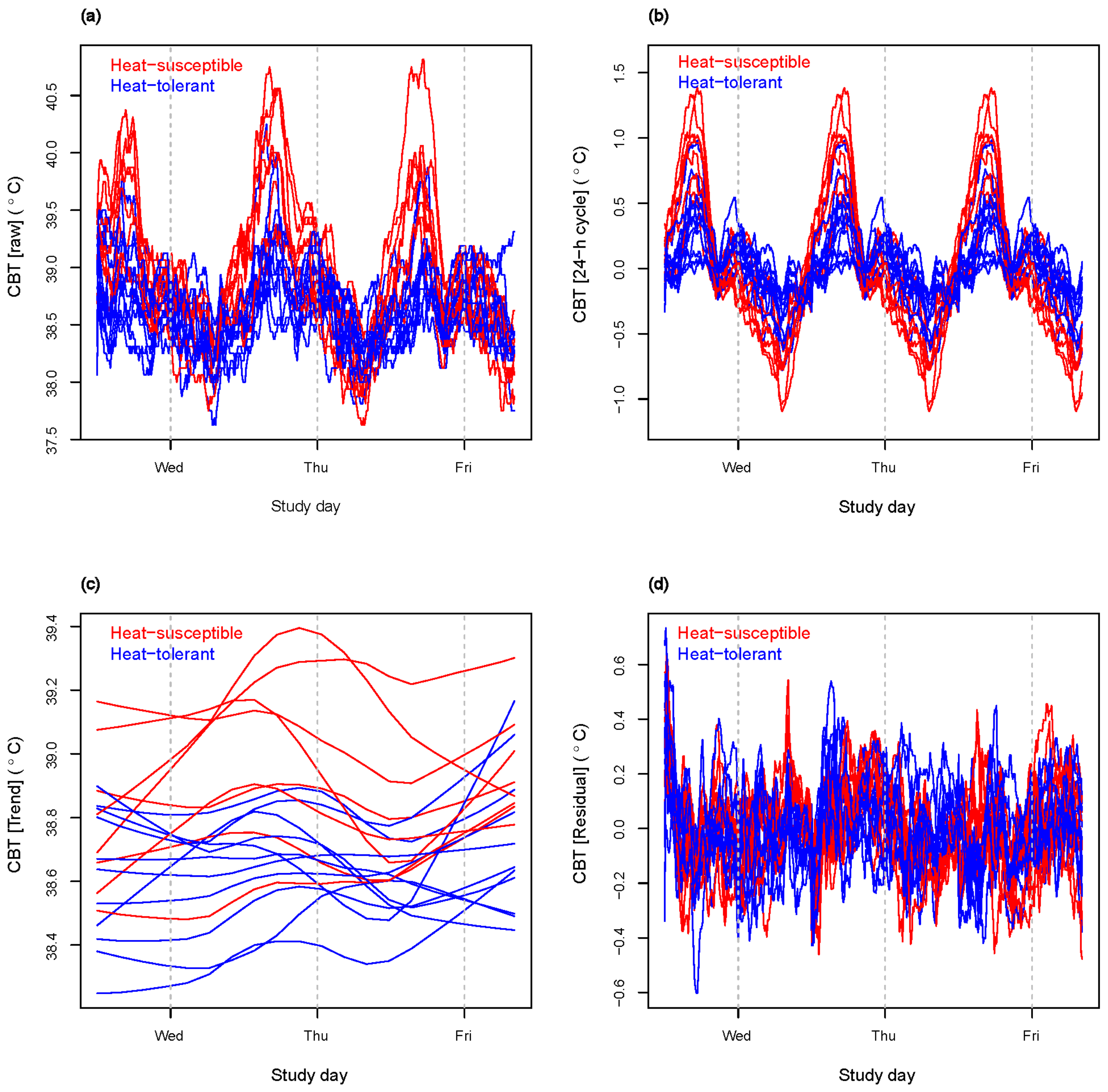

Supplementary Figure S5. Decomposition of 68-h raw core body temperature (CBT) time-series data into deviations for 24-h cycle, trend, and residual deviations. Each heifer is shown as a separate line, showing heat-susceptible (red) and heat-tolerant (blue) individuals.

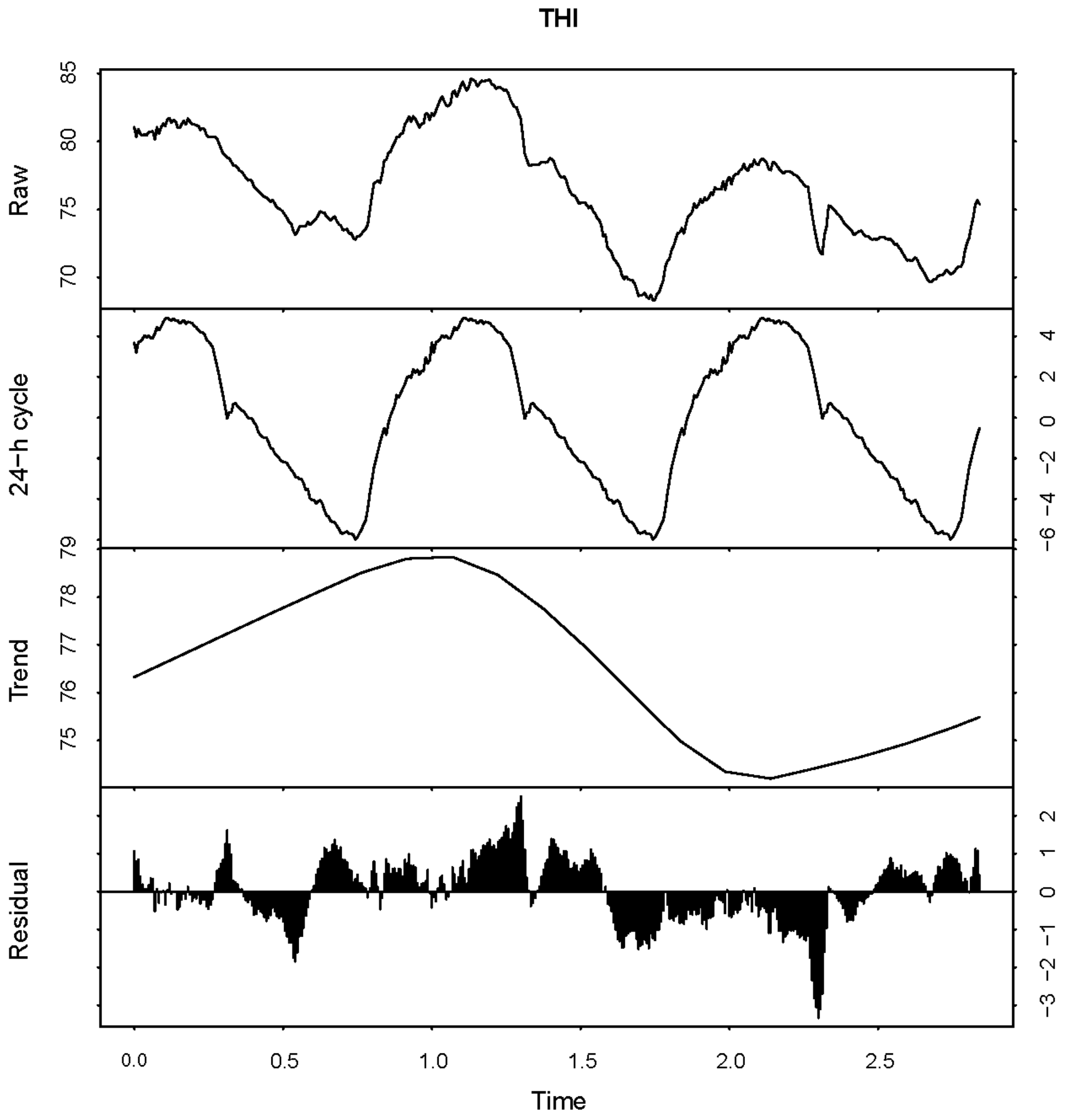

Supplementary Figure S6. Decomposition of 68-h raw temperature humidity index (THI) time-series data into deviations for 24-h cycle, trend, and residual deviations.

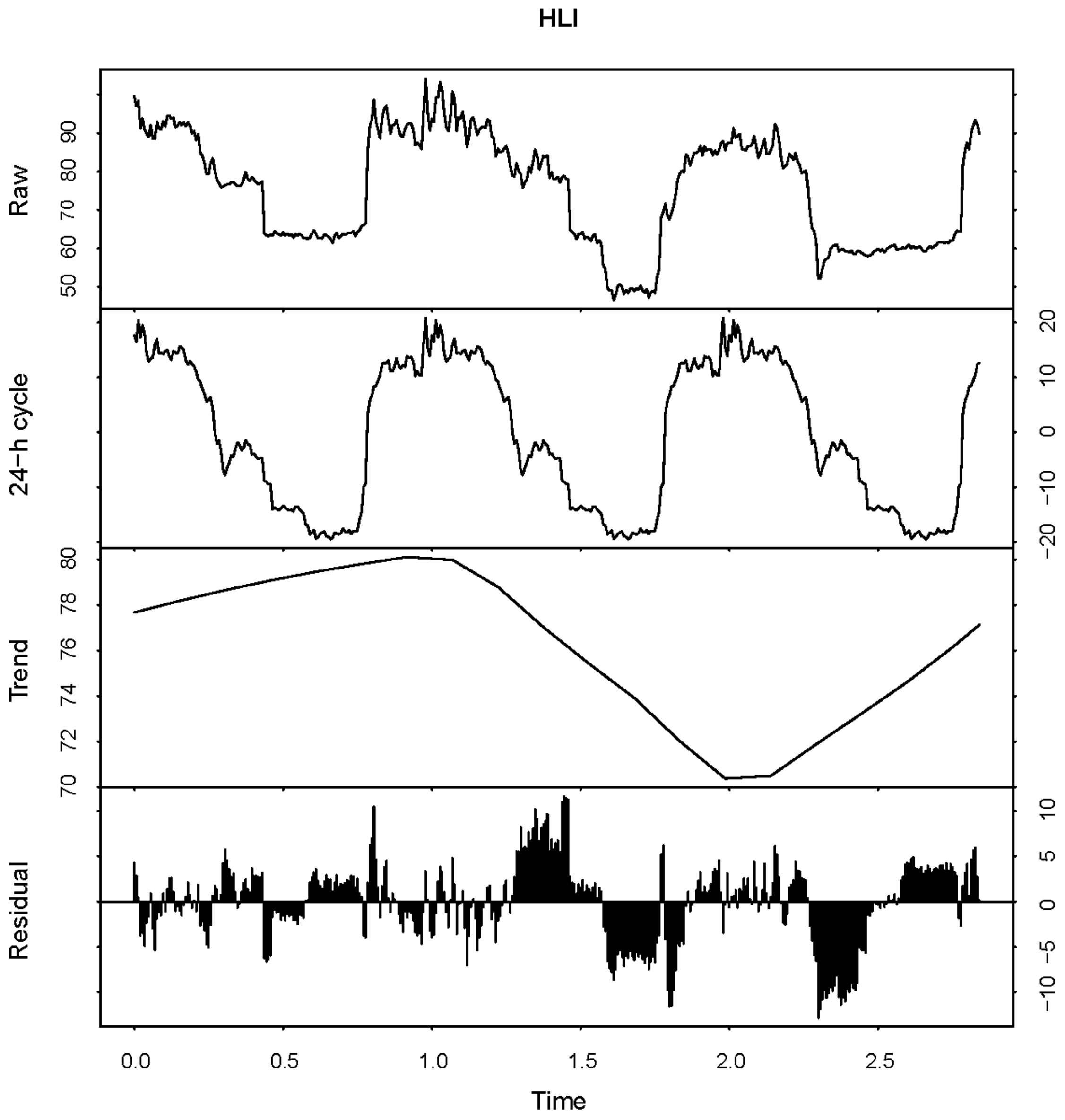

Supplementary Figure S7. Decomposition of 68-h raw heat load index (HLI) time-series data into deviations for 24-h cycle, trend, and residual deviations.

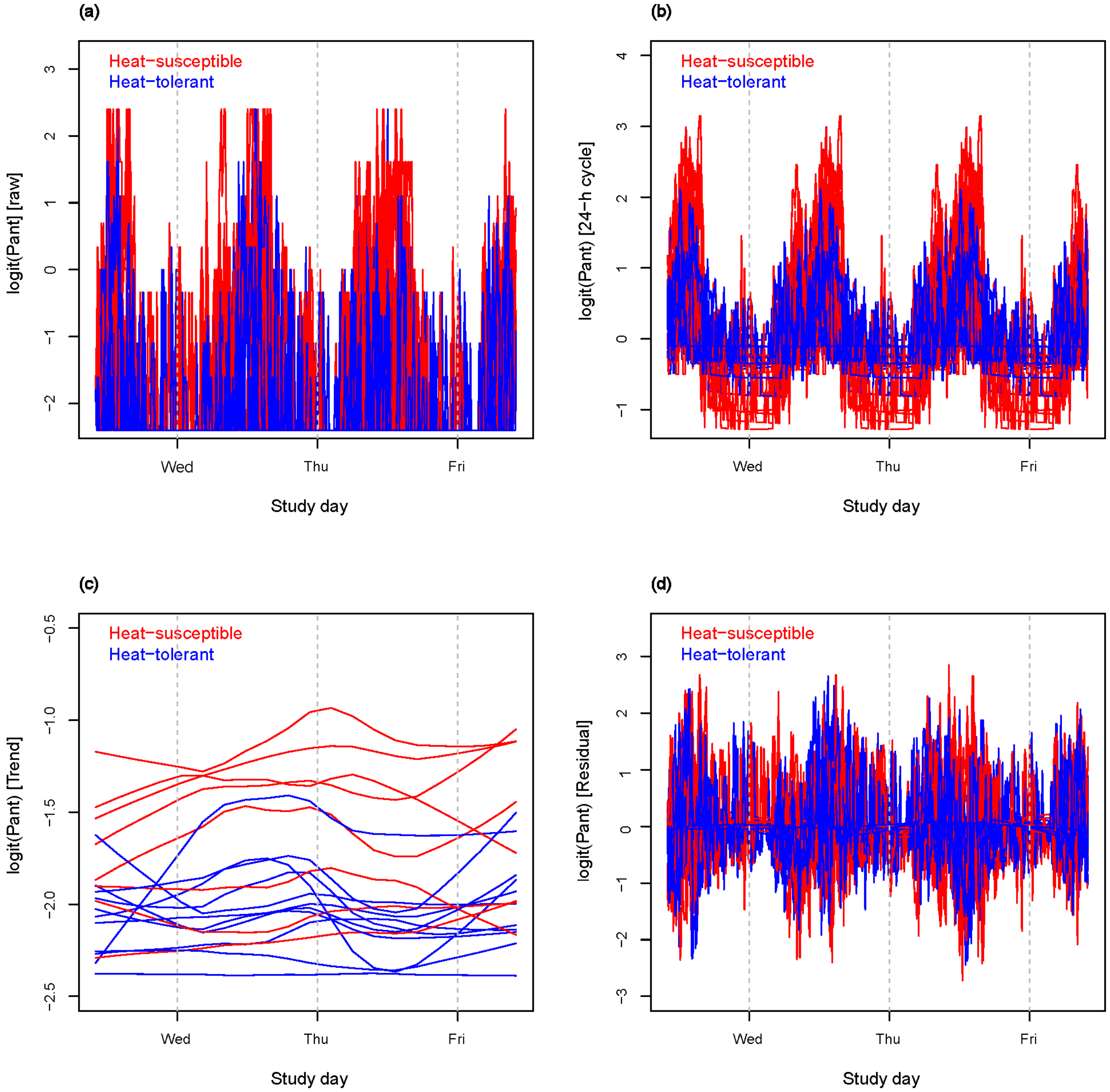

Supplementary Figure S8. Decomposition of 68-h raw panting (logit(Pant)) time-series data into deviations for 24-h cycle, trend, and residual deviations. Logit function was used as panting was recorded at minute-level as a binary (Yes = 1 and No = 0) outcome. Each heifer is shown as a separate line, showing heat-susceptible (red) and heat-tolerant (blue) individuals.
